# Improving Cognitive Workload in Radiation Therapists: A Pilot EEG Neurofeedback Study

**DOI:** 10.3389/fpsyg.2020.571739

**Published:** 2020-12-03

**Authors:** Alana M. Campbell, Matthew Mattoni, Mae Nicopolis Yefimov, Karthik Adapa, Lukasz M. Mazur

**Affiliations:** ^1^Department of Psychiatry, School of Medicine, The University of North Carolina at Chapel Hill, Chapel Hill, NC, United States; ^2^Department of Radiation Oncology, School of Medicine, The University of North Carolina at Chapel Hill, Chapel Hill, NC, United States

**Keywords:** neurofeedback, EEG, microstates, cognitive workload, radiation oncology, burnout, NASA-TLX, alpha/theta

## Abstract

Radiation therapy therapists (RTTs) face challenging daily tasks that leave them prone to high attrition and burnout and subsequent deficits in performance. Here, we employed an accelerated alpha-theta neurofeedback (NF) protocol that is implementable in a busy medical workplace to test if 12 RTTs could learn the protocol and exhibit behavior and brain performance-related benefits. Following the 3-week protocol, participants showed a decrease in subjective cognitive workload and a decrease in response time during a performance task, as well as a decrease in desynchrony of the alpha electroencephalogram (EEG) band. Additionally, novel microstate analysis for neurofeedback showed a significant decrease in global field power (GFP) following neurofeedback. These results suggest that the RTTs successfully learned the protocol and improved in perceived cognitive workload following 3 weeks of neurofeedback. In sum, this study presents promising behavioral improvements as well as brain performance-related evidence of neurophysiological changes following neurofeedback, supporting the feasibility of implementing neurofeedback in a busy workplace and encouraging the further study of neurofeedback as a tool to mitigate burnout.

## Introduction

Radiation therapy therapists (RTTs) show high attrition as well as high rates of burnout (Probst et al., 2012; Schofield et al., 2012; Halkett et al., 2017; Singh, 2017; Guerra and Patricio, 2018), which results in deficits in care effectiveness, job satisfaction, patient safety and satisfaction, and overall organizational success (Shanafelt and Dyrbye, 2012). Thus, finding a way to reduce job stress and consequently prevent burnout is an important priority, with organizations making investments to improve the wellness of the workforce (Ishmaila et al., 2018).

Neurofeedback (NF) is emerging as a potential solution to address negative consequences of stress and burnout and warrants examination of potential behavior and brain related benefits (Fronda et al., 2019). NF is a form of biofeedback focused on modifying brain activity supporting a broad range of complex behaviors and thoughts, including stress, focus, and cognition through directed and effortful self-regulation of neural oscillatory activity of the electroencephalogram (EEG) (Sitaram et al., 2017). Electroencephalogram activity may be divided into canonical frequency bands, including theta (4–8 Hz) which reflects cognitive effort and is directly tied to load, and alpha (8–12 Hz) which has been studied with stress and arousal (Klimesch et al., 2005). Both theta and alpha bands are frequent targets for NF interventions (Marzbani et al., 2016b). In NF training, participants use visual or auditory feedback of a specific EEG frequency band power to modify their neural oscillatory activity in attempt to alter functions associated with the frequency band. For example, participants may use NF to increase their theta power, resulting in improvements in mood and cognitive performance (Gruzelier, 2014).

Neurofeedback has been effective at alleviating symptoms of anxiety and depression (Hammond, 2005; Cheon et al., 2016; White et al., 2017), improving mood (Raymond et al., 2005; Choi et al., 2011), reducing symptoms of burnout (Mazur et al., 2019), and decreasing stress (Wook Weon et al., 2008; Hafeez et al., 2019). It has also recently been applied to healthy individuals in high stress positions. For example, NF was tested among high-functioning professionals in managerial positions, who reported decreased perceived stress levels and increased concentration and focus (Fronda et al., 2019). In a healthcare setting, studies suggest that NF can be used to teach physicians and nurses to enter into a state of relaxed alertness and improve their wellbeing (Dunham, 2019). Additionally, previous research found NF to improve cognitive workload during working memory tasks (Mazur et al., 2017). Furthermore, NF trainings have been associated with improved performance on complex tasks (Vernon et al., 2003; Escolano et al., 2011). Importantly, NF has consistently been linked to post-intervention changes in EEG activity (Gruzelier, 2014), suggesting that NF learning altered brain physiology along with producing cognitive-behavioral improvements.

One way to measure improvements in cognition is through experienced workload, a measure of the amount of resources needed to complete a task (Gevins and Smith, 2003). Both theta activity and cognitive performance have been linked to workload (Funke et al., 2013; Fallahi et al., 2016). A high workload can impart risk for performance and safety related errors (Michtalik et al., 2013), and is related to burnout (Greenglass et al., 2001). Thus, elevated workload could contribute to RTT errors and burden. For RTTs, professional, emotional, and psychological demands are high, all of which likely contribute to an increased cognitive workload. When workload capacity is reached and no additional resources can be recruited, individuals begin to exhibit symptoms of emotional fatigue, stress, and depression (Golonka et al., 2017) which can produce a vicious cycle and lead to deterioration of mental health and quality of performance. Thus, if NF can lessen cognitive workload, it could be useful in both preventative and treatment methods to mitigate these harms.

However, there is a high degree of variability in the recommendations for NF session protocols (Marzbani et al., 2016b), a lack of knowledge as to the influence of NF training in a high-stress field, such as radiation therapy, and a practical issue in that many NF protocols take a substantial amount of time to learn and employ. Very recently, a collaboration produced guidelines for NF study design that should assist the field in addressing these issues (Ros et al., 2020). Still, it remains unknown if NF is feasible as a tool in busy workplace settings, such as a hospital, to decrease stress, and improve performance. Thus, we designed a study to determine if a 3-week NF protocol in a hospital setting could be (a) successfully implemented, (b) learned, and (c) produce measurable behavioral and neurophysiological changes in RTTs. To that end, we analyzed pre- and post-NF EEG and microstate activity during a computerized performance test (CPT) as measures of RTT neurophysiological changes to indicate RTT ability to learn NF. Although changes of EEG activity have been well established following NF (Gruzelier, 2014), microstate analysis is significantly less researched with NF and was thus of particular interest as an additional measure of modification of brain states. We also conducted pre- and post-NF assessments of cognitive workload through the National Aeronautics and Space Administration Task Load Index (NASA-TLX; Hart and Staveland, 1988). Thus, cognitive performance during the CPT and cognitive workload were used to compound EEG results as subjective measures of benefits relating to NF. We predicted that if the protocol could be learned, as shown by neurophysiological alterations measured by EEG, we would observe reductions in workload and improvements in performance. A notable limitation of the study is a lack of a control group, which prevents specific causal connection of NF to changes in EEG or cognitive performance. However, this study’s primary importance is examining the feasibility and potential benefits of an accelerated NF protocol in a hospital setting.

## Methods

Twelve (six male) healthy adult radiation therapists were recruited as participants from the Radiation Oncology Department at The University of North Carolina at Chapel Hill. All participants gave informed consent in accordance with the University Institutional Review Board prior to their participation. Participants completed an intake visit with a trained clinician, in which they answered questions about their general wellbeing, stress, sleep, and areas of possible concern. Pre- and post-NF intervention, participants completed a CPT while recorded by EEG.

### Neurofeedback Protocol

The NF intervention consisted of eight NF sessions, each 28 min in length, over a 3-week period targeting cortical alpha/theta/beta activity in both temporal lobes at location C5 and C6 (from the international 10–20 system of electrode placement (Silverman, 1963). Each session started with instructions for the RTTs to remain relaxed and still for approximately 20–40 s as BrainPaint^®^ software gathered baseline measures for the targeted frequencies. The EEG biofeedback methodology we employed utilized 24 min alpha theta training with Pz alpha suppression that was based on the Scott and Kaiser modification of the Peniston protocol (Scott et al., 2005). Immediately following the alpha theta training we administered 4 min of temporal lobe theta/beta ratio training at C5–C6 (Keith et al., 2015). All training was performed on the BrainPaint platform.

The frequency range for alpha was 8–11 Hz and for theta it was 5–8 Hz with the active sensor placed at Pz. Rewards were 40–60% above threshold for theta. All inhibits were maintained between 10 and 20% below threshold. When the rewards and inhibits remain outside these percentages for more than a minute the thresholds automatically adjust to return within those desired ranges. The initial portion of the sessions were used to train down (peak to peak) alpha amplitudes augmenting theta, until there was “crossover.” This was defined as the point at which the alpha amplitude drops and the theta amplitude crosses over it. Alpha amplitude attenuation and subsequent amplitude increases were both rewarded between 50 and 70%. Subsequent to the first achievement of crossover, both alpha and theta frequencies were augmented. Excess EEG amplitudes in the range of 15–30 Hz and 2–5 Hz were inhibited between 10 and 20% above threshold. This was intended to reduce muscle tension, to quiet the mind yet remain awake. Each alpha-theta session began with the subject sitting in a chair at a 45 degree angle with eyes closed. The active electrode was placed at Pz with a left-ear reference (A1). The right earlobe grounded. Two distinct tones were employed for alpha and theta reinforcement, with the higher pitched sound used to index the higher-frequency alpha band and a lower pitch for theta. At the start of each alpha theta session, the nf research personnel spent 3–5 min gathering OK responses from the subjects after the machine played four segments of a generic guided visualization that dealt with imagery of wellbeing, specific reaction or behavior change scenes, and a positive recollection.

The 4-minute theta/beta ratio training had sensors placed at C5 and referenced to C6 grounded at A1 (left ear). Sensor impedance was tested and kept below 5 k ohms with the BrainMaster Atlantis two with a 256 Hz sampling rate. The reward conditions (sounds and graph) informed participants of increased EEG peak to peak amplitude in frequencies in the ranges between 15 and 18 Hz. The inhibit conditions stop all sounds and cause a graph to elevate in the vertical direction for frequencies in the 1–12 Hz and 22–30 Hz ranges. Frequencies outside of these ranges did not influence feedback. Segments of EEG that contained noise exceeding 100 μV were classified as artifacts (e.g., movement or muscle) and produced a beep sound and purple EEG. Participants received a 20 s break after every 2 min of training. Thresholds were adjusted through automation in a way that if the participant maintained the reward band above the threshold between 60 and 80% of the time during at least 0.25 s while the two inhibit bands under the threshold for 10–20% of the time, feedback was delivered. When the rewards and inhibits remain outside these percentages for more than 30 s the thresholds automatically adjusted to be back within the desired ranges. Whenever participants could maintain the reinforced EEG frequencies above the threshold while reducing the amplitude of inhibit frequencies below threshold additional sounds were presented and the graph would vertically drop until it was at its lowest of six possible positions. Thus, subjects were instructed to keep the sounds playing and cause the graph to drop or remain at its lowest of six positions (Keith et al., 2015).

Participants averaged approximately 400 s (with 420 s being the theoretical maximum) of hold time per protocol by the third session indicating that they were engaged during the intervention.

### Behavioral Measures: CPT and NASA-TLX

**FIGURE 1:**
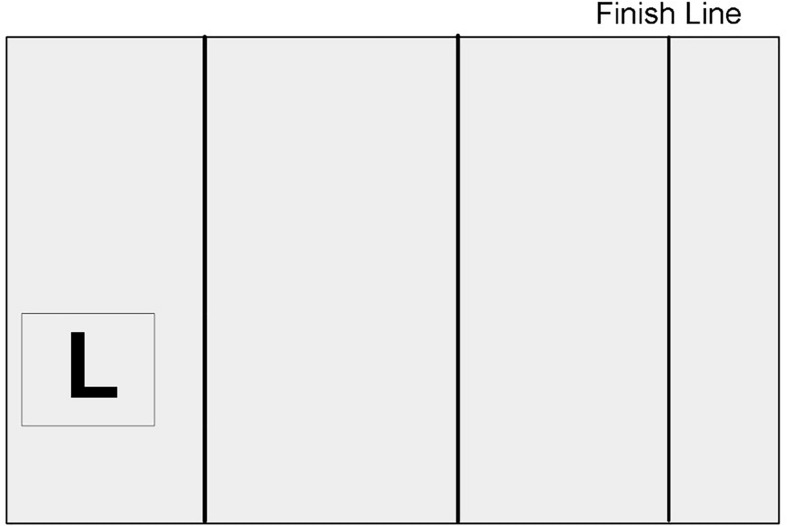
CPT Illustration. The box on the left showed either “L,” indicating a left button press, “R,” indicating a right button press, or “P,” indicating pass. The box moved one pixel to the right following a correct response, or two pixels to the left following an incorrect response. Reaching the finish line required 600 correct responses if no mistakes were made. R = right, P = pass.

A BrainPaint^®^ CPT was administered pre- and post-NF intervention using 2.7 GHz quad core PC. This test acquired raw EEG at 256 Hz, with impedances below 5 kΩ. Raw EEG data were saved as standard EDF format for later analysis. Participants responded to go/no-go style stimuli. They were presented with three letters (L,R, and P; Figure 1), two of which required a response and one required the inhibition of a response. RTTs held a two-button mouse in two hands with their left and right thumbs over the two respective mouse buttons. When RTTs saw the letter “L” they were to press the left and when they saw an “R” they were to press the right button. When they saw the “P” they paused for that individual’s current average response time until the next letter appeared. All letters were generated randomly. Any response instantly generated a beep and presented the next letter within 30 ms. A correct response advanced the two-inch letter one pixel to the right toward a visible finish line. An incorrect response moved the letter ten pixels to the left away from the finish line. The test required 600 correct responses to cross a finish line, if no errors were made. The actual game required a pretest of 100 correct responses. RTTs were asked to complete the game as quickly and as accurately as they could. The task required concentration, attention to detail, and motor control. Following each CPT, RTTs completed the NASA-TLX instrument to assess workload (Hart and Staveland, 1988). Workload represents the overall cost incurred by a human operator to achieve a particular level of performance (Hart and Staveland, 1988). Across disciplines, the NASA-TLX instrument has been validated and widely used to subjectively measure workload (Hart, 2006). In summary, the NASA-TLX is based on a multi-dimensional rating procedure that considers six dimensions (mental, physical, and temporal demands; frustration, effort, and performance) to yield a global workload score between 0 and 100.

### Neurophysiological Measures: EEG and Microstate Processing

Electroencephalogram was recorded pre- and post-NF intervention during the CPT. EEG files were imported to and analyzed with EEGLAB (Delorme and Makeig, 2004). A high-pass filter was used to remove frequencies below 1 Hz and artifact subspace reconstruction (ASR; *clean_rawdata*) was used to estimate data from highly variant or missing sources (Chang et al., 2018). Power spectra for pre- and post-NF intervention recordings for each participant were generated by fast Fourier transforms within-subject changes across the canonical frequency bands, from 1 to 45 Hz, and we specifically extracted and examined average power values via *newtimef* for theta (4–8 Hz) and alpha bands (8–12 Hz).

Microstates were analyzed in microstate toolboxes developed for EEGLAB (Michel and Koenig, 2018; Poulsen et al., 2018). One thousand global field power (GFP) peaks were used for microstate segmentation using a modified K-means algorithm and sorted by global explained variance. Segmentations were completed in 50 repetitions with a maximum of 5,000 iterations and a convergence relative threshold of 1 × 10^–6^. Four active microstate maps were then back-fit onto the EEG data and temporally smoothed to reduce noise and unstable topographies, leaving the time-series with microstate labels at each point. Finally, mean GFP values and microstate durations were extracted for each microstate. Due to limitations of a two-channel EEG, analysis focused on the main microstate cluster. One participant was excluded due to microstate cluster activity that exceeded 2.5 standard deviations from the mean.

### Statistical Analysis

Separate linear mixed models were used to analyze each of the neurophysiological measures and behavioral measures. Each mixed model was fit with maximum likelihood and had pre/post included as a fixed term, and allowed for individual variation at baseline (intercept, pre-NF) and in change from pre to post-NF (slope, pre to post-NF), thus responses were nested within participant. All data were analyzed in R with RStudio 1.2.5003 (R Core Team, 2019) using the lme4 package (Bates et al., 2015) *t*-tests report on the effects nested within subjects with a significance level set at 0.05.

## Results

All 12 participants completed all NF sessions, the results for whom are reported below and summarized in Table 1.

**TABLE 1:** Behavioral and neurophysiological measures.

Measure	Pre (Mean, SD)	Post (Mean, SD)	*t*-test (*t, p*)
NASA-TLX workload	24.3, 12.25	19.7, 13.3	**6.05, *p* < 0.01**
CPT response time (ms)	640, 41	619, 56	**2.4, *p* = 0.036**
CPT errors	15, 11	20, 16	−1.1, *p* = 0.31
EEG alpha power (μV)	−3.1, 1.4	−2.2, 1.4	**2.36, *p* = 0.038**
EEG theta power (μV)	−2.4, 2.3	−0.9, 1.9	1.99, *p* = 0.072
Microstate GFP (μV)	19.6, 4.0	17.1, 5.8	**2.5, *p* = 0.032**

*Bold indicates statistical significance.*

### Behavioral: Cognitive Performance and Load

Response time during CPT, from the pre-test (*M* = 640 ms, *SD* = 41 ms) and post-test (*M* = 619 ms, *SD* = 56 ms) sessions indicated a significance decrease in response time [*t*(11) = 2.4, *p* < 0.04]. However, for errors made during CPT, the pre-test (*M* = 15, *SD* = 11) and post-test (*M* = 20, *SD* = 16) showed no significant difference [*t*(11) = −1.1, *p* > 0.05].

There was a significant reduction in NASA-TLX scores following the NF intervention [*t*(11) = 6.05, *p* < 0.01, *B* = −8.91] suggesting an eight point reduction following NF, indicating a decrease in subjective cognitive load.

### Neurophysiological: EEG and Microstate Analysis

**FIGURE 2:**
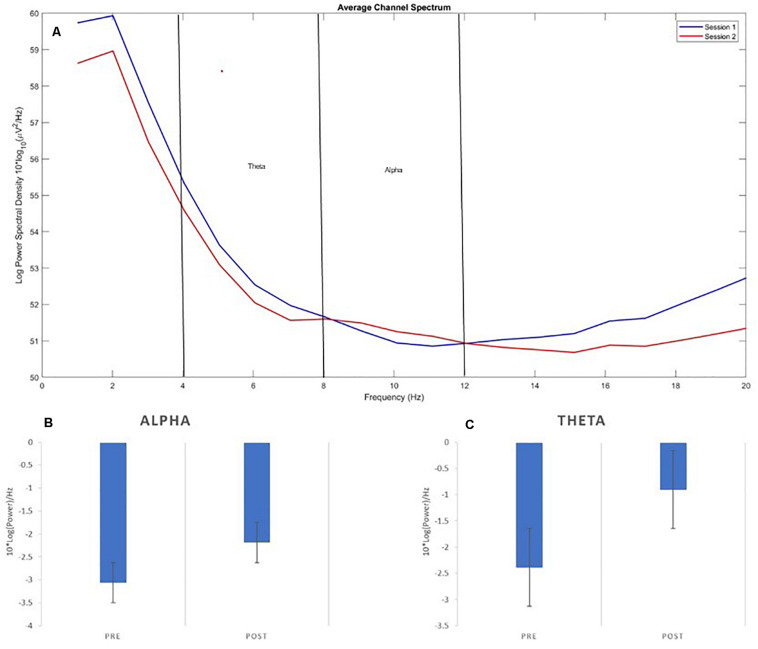
EEG Power. **(A)** Displays the average power spectrum distribution for the two EEG channels pre- and post-neurofeedback, **(B)** Average Alpha (8–12 Hz), and **(C)** Theta (4–8 Hz) pre- and post-neurofeedback.

Electroencephalogram event-related spectral perturbation was extracted across frequencies and averaged for theta and alpha frequency bands. There was a reduction in the negativity of the mean alpha power following NF training [*t*(12) = 2.36, *p* = 0.04], see Figure 2. Theta activity showed greater between subject variability and a marginal change from pre- to post-NF intervention [*t*(11) = 1.99, *p* = 0.072], see Figure 2. This suggests that theta moderately increased, while alpha decreased (in change from baseline) over the course of the protocol.

The primary microstate cluster (MS1) was extracted and compared across participants from pre- to post-NF intervention. There was a significant decrease in GFP at the main microstate cluster following NF [*t*(11) = 2.50, *p* = 0.03].

### Combining Behavioral and Neurophysiological Measures

We calculated the change in each neurophysiological and behavioral measures (NASA-TLX, CPT-response time) from pre- to post- and tested the correlations between the observed neurophysiological and behavioral changes. Indeed, the change in NASA-TLX correlated with the change in alpha power (*r* = 0.66, *p* = 0.01) but not with theta power (*r* = −0.41, *p* = 0.16) or microstate GFP (*r* = −0.27, *p* = 0.37). However, the CPT task response time correlated with only microstate GFP (*r* = 0.61, *p* = 0.025) but not theta (*r* = −0.44, *p* = 0.12) or alpha (*r* = 0.44, *p* = 0.12) power.

## Discussion

Radiation therapy therapists work environments leave them prone to burnout and consequential limitations in performance and mood. NF is a potential tool to address these issues as it can be implemented in a busy medical setting and has shown promise to improve cognitive performance and alter neurophysiology. Here, we studied a 3-week NF protocol to determine the protocol’s feasibility and extent to which 12 RTTs could learn, and benefit from, a course suited for the busy schedules of RTTs. We report a significant reduction in self-reported cognitive load (*p* < 0.01) along with decreased response time during the cognitive test (*p* < 0.04) following the NF protocol. These behavioral findings are supported by neurophysiological alterations, specifically a significant reduction in alpha desynchrony (*p* < 0.04), a significant decrease in microstate GFP (*p* < 0.03), and a marginal increase in theta power (*p* < 0.07) following NF. Together, these behavioral and neurophysiological results suggest that accelerated NF is a protocol that can be learned and implemented in a busy setting and is linked to cognitive workload improvements.

Alpha and theta activity are consistently shown to support cognitive and executive functioning, particularly in stressful situations (Subhani et al., 2013). Here, we found a decrease in alpha desynchrony and, to some degree, an increase in theta activity from the initial to final session of the accelerated NF training protocol during a cognitive performance test. These EEG results are consistent with the alpha/theta protocol (Egner et al., 2002) and have been shown by other studies to be related to improvements in mood (Peninston and Kulkosky, 1989) and default mode network connectivity (Imperatori et al., 2017). Thus, EEG results served as an indication that the NF could be learned by RTTs in a manner consistent with improving cognitive load. Moreover, the reported behavioral improvements in cognitive workload were associated with the change in alpha power. This could reflect the role of alpha activity in both focusing on a task and preventing interfere (Klimesch, 2012). While we cannot conclude that NF specifically caused these changes, previous studies have supported the efficacy of NF in altering EEG relative to control (Vernon et al., 2003; Raymond et al., 2005).

Further supporting this notion, we found a small decrease in GFP for the main microstate cluster following NF. While microstate analysis was limited by the two-electrode EEG system, this result indicates that NF was associated with a more efficient brain state during the cognitive performance test, supporting the idea of improved workload. Microstate analysis allows one to track changes in brain states, similar to the objective of NF, and was used here to support EEG and cognitive-behavioral results. This result may reflect more efficient activity supporting CPT performance because of learning. To our knowledge, this is the first study to examine how alpha/theta NF affects microstates. Indeed, the observed significant relationship between the improvements in CPT behavioral results and the microstate GFP support this idea. This result is encouraging and warrants further study for how NF can alter microstates, with another exciting study identifying the feasibility of microstates as the basis for an alternate NF protocol (Diaz et al., 2016).

As with any study, there were several limitations. Primarily, there was no control group. Thus, it is unlikely but possible that reported power and microstate modifications post-NF intervention were due to other factors (e.g., learning/familiarity with CPTs). However, it is worth again noting that prior NF studies do not report significant improvements in EEG signals in control groups (Vernon et al., 2003; Raymond et al., 2005). Additionally, the primary goal of the current study was to investigate the feasibility of an accelerated NF protocol in this high-aptitude, busy work schedule. Additionally, the sample size was small (*n* = 12) due to the nature of this feasibility study within a hospital, limiting generalizability. Furthermore, the study would have benefited from additional measurements related to cognitive performance and standardized measurements of mood. Specifically, tasks varying in cognitive demand such as the n-back would provide useful information to the extent to which NF can improve performance, and a standardized mood questionnaire could better address how the NF mitigated the effect of burnout. Future studies should also consider other physiological measurements, such as heart rate variability, as this would provide well-researched physiological data to support brain-related results.

In sum, positive findings from cognitive-behavioral tests and neurophysiological measures of EEG power and microstate GFP converge to suggest both feasibility and associated practical benefits of implementing a NF intervention into a busy healthcare work schedule, particularly to combat issues associated with burnout. On a larger scale, this study contributes information regarding the use of an accelerated protocol in the workplace and microstate analysis to the growing NF literature.

## Data Availability Statement

The raw data supporting the conclusions of this article will be made available by the authors, without undue reservation.

## Ethics Statement

The studies involving human participants were reviewed and approved by The University of North Carolina IRB. The patients/participants provided their written informed consent to participate in this study.

## Author Contributions

AC, MM, and MY processed and analyzed the EEG data. KA and LM processed behavioral data. LM conceptualized the study. AC conducted the statistical analysis. AC and MM drafted the manuscript. All authors contributed to the article and approved the submitted version.

## Conflict of Interest

The authors declare that the research was conducted in the absence of any commercial or financial relationships that could be construed as a potential conflict of interest.
